# Azhe'é Bidziil (Strong Fathers): Study Protocol for the Pilot Evaluation of an American Indian Fatherhood Program to Improve the Health and Wellbeing of Diné (Navajo) Fathers

**DOI:** 10.3389/fpubh.2021.790024

**Published:** 2022-02-10

**Authors:** Jennifer Richards, Tiffani Begay, Rachel A. Chambers, Hima Patel, Justin Mayhew, Joshuaa Allison-Burbank, LeDaniel Gishie, Nolan Tsingine, Julius Badoni, Leander Staley, Bradlin Harvey, Alicia Tsosie, Marissa Begay, Kristin Mitchell, Lauren Tingey

**Affiliations:** Johns Hopkins University Bloomberg School of Public Health, Baltimore, MD, United States

**Keywords:** fathers, fatherhood, American Indian, Indigenous, intervention, Native

## Abstract

**Introduction:**

Considering the critical role that American Indian and Alaska Native (Native) men play in family and child health, there is an urgent need to collaborate with Native communities in developing interventions and policies to improve Native men's health status. This study aims to address a significant gap in research by designing and implementing a culturally grounded health promotion program to increase economic stability, promote positive parenting, and build healthy relationships among Native fathers. The Azhe'é Bidziil (“Strong Fathers”) study protocol, developed in response to community advisory board feedback, illustrates a community-engaged approach to developing and implementing a fatherhood program in two Diné (Navajo) communities.

**Methods/Analysis:**

Azhe'é Bidziil was adapted from three evidence-based interventions developed in collaboration with Native communities. Intervention lessons were iteratively reviewed by a tribal working group to ensure that the content is culturally appropriate and relevant. A pre-post study will assess feasibility, acceptability, and satisfaction with the Azhe'é Bidziil intervention, as well as short-term impacts on positive parenting, economic stability, and healthy relationship outcomes. The intervention is composed of 12 weekly group sessions conducted with fathers (*n* = 750) that focus on developing knowledge and skills for positive father involvement, economic stability, and healthy relationships. Lesson content includes: honoring our roles as fathers, building healthy relationships, understanding the impact of historical trauma, goal-setting, and budgeting basics. Each of the 12 group lessons, consisting of 8–12 participants per group, last approximately 2 h. Eligible fathers or father figures are age ≥18 years, live within 50 miles of the participating Diné communities, and must be caregivers of at least one child ≤ 24 years. The outcomes for this study are acceptability, feasibility, and satisfaction with the intervention, as well as father involvement, quality of (co-) parenting communication, healthy relationships, fathers' engagement and communication with their children, protective factors (e.g., cultural connectedness and educational/career aspirations), and economic empowerment and stability. Participants will complete an outcome assessment at pre- and post-intervention (12 weeks later).

**Discussion:**

This study protocol presents one of the few evaluations of a fatherhood intervention to increase economic stability, promote positive parenting, and build healthy relationships among Native fathers in rural tribal communities. Such a study is sorely needed to address the health disparities perpetuated by social and Indigenous determinants of health that Native men experience today. If proven efficacious, this pre- post-study will inform a large scale randomized controlled trial to evaluate intervention impact, and if proven efficacious may be disseminated widely in tribal nations. Study findings may also deepen our understanding of peer mentoring, Native men's health status, involvement with their children, co-parenting relationships, family relationships, cultural connectedness, and economic status. The data collected may also inform strategies to ensure acceptability, feasibility, and satisfaction of an intervention designed specifically for Native fathers.

## Introduction

American Indian and Alaska Native (AIAN, Native) societies have been at the forefront of resistance against decades of land theft, colonization, and attempts at assimilation since time immemorial (1, 2). It is imperative to understand Native history, specifically the strength and health of Native men, as it stands in stark contrast to the health disparities that AIAN men face today. Native men have some of the highest rates of chronic diseases including diabetes (3), chronic liver disease (4), cardiovascular disease (5), and excessively high death rates for prostate, liver, and colon cancers (6). Despite projected national gains in life expectancy over the next few decades, Native men still have a disproportionately higher mortality rate compared to non-Hispanic white men (7) and are expected to live 6.6 years less than Hispanic/Latino men and 4.4 years less than non-Hispanic White men (8).

Many of the health disparities experienced by Native men today can be attributed to structural and social determinants of health that affect Native communities. Structural and social determinants of health are environmental conditions “where people are born, live, learn, work, play, worship, and age that affect a wide range of health, functioning, and quality-of-life outcomes and risks” (9). Inequities in social determinants of health have been linked to poorer health outcomes and premature mortality across racial and ethnic groups (7, 10). Examples of these structural and social determinants of health include: safe housing and roads; access to healthy food; experiences with racism; access to quality education, job opportunities, and income (9). Native peoples experience disparities across these varying structural and social determinants of health, including high rates of unemployment (11) which is over 40% in some tribal communities (12), and over twice the poverty rate of non-Hispanic Whites (NHW) (11). Native people are less likely to attain a bachelor's degree than the general US population (21 vs. 37%) (11) and are often born into single-headed households (13). In 2019, over half (52%) of Native children were living with one parent (13) and in 2019, nearly 70% of Native babies were born to unmarried women (14).

Native men's health status has also been uniquely impacted by Indigenous determinants of health, which extend beyond the social and structural determinants described (15). These determinants, which are all influenced by colonialism, include historical trauma, federal assimilation policies, and racism, as well as protective factors such as cultural teachings, connection to land, sovereignty, language, and spirituality (15). For example, forced relocation of AIAN children into boarding schools disrupted Native parenting practices, cultural connectedness, kinship networks, and traditional language learning (16–19). This disruption to Native livelihood has manifested through the generations as substance abuse, domestic violence, mental health disorders, suicide, and poverty (16, 17). Assimilation policies were especially devastating for Native men as the forced removal of Native boys from their positive male role models disrupted intergenerational warrior teachings (20). Thus, scholars cite these policies as major contributing factors to the high rates of substance use (16, 18, 19, 21), parental absence (22), poverty (19, 23), accidents, suicide, violent victimization (5), and incarceration experienced by Native men today (24, 25).

With an enrolled population of approximately 399,500, the Navajo Nation is the largest tribe in the country (26). The Navajo Nation is also the largest AIAN land base in the United States, covering 27,425 miles of rural land in New Mexico, Arizona and Utah (27). Diné (Navajo) men experience the same health disparities and inequities in aforementioned structural and social determinants of health as AIAN men across the US. From 2006 to 2009, Diné men residing on the Nation had a slightly higher age-adjusted mortality rate than US White males in 2009 (876.7 vs. 875.9, respectively) (28, 29). For this same time period, the top 5 causes of Diné male mortality were largely preventable conditions and included: unintentional injury, diabetes, suicide, alcohol dependence syndrome, and assault (29).

The Johns Hopkins Center for American Indian Health (JHU; “the Center”) and the Navajo Nation have a 30-year community-academic partnership that addresses health disparities across the life span. Over the decades long partnership, the Center had implemented various family and child health interventions but had yet to implement one specifically targeting Native fathers. Community advisory boards, composed of organizational partners, health providers, traditional healers, and community members, voiced the need for a fatherhood empowerment intervention to holistically address family and community health. The formative work for the current protocol began as early as 2010 when a small (*N* = 87) descriptive study looking at fatherhood roles and substance use also identified risk factors to target for a future fatherhood intervention, including high unemployment status and low involvement in childcare (18). Recently, female youth participants in another study reinforced the supportive role that fathers and male relatives served during their rite of passage cultural events (30). Overall, with preliminary data and strong community support, the Navajo Nation's longstanding partnership with the Center provided a strong foundation to develop and pilot a fatherhood health intervention.

Considering the critical role that Native men play in family and child health, there is an urgent need to collaborate with tribal communities in identifying programs and policies to improve Native men's health status and increasing family involvement. Fatherhood involvement has a positive relationship with early childhood development (1) and has been shown to positively impact children's social and emotional health, as well as academic success during critical developmental years (31–34). This positive impact is especially pronounced for lower income children and for those who lack access to high quality educational systems, including Native youth (35–37). This finding is particularly relevant since Native children experience poverty and school dropout rates at higher rates than other racial and ethnic groups, as previously described. Recent studies have also demonstrated the positive impact of fatherhood involvement on child development at all stages (37) child academic performance (38) and overall child wellbeing (39).

Despite growing research on the benefits of fatherhood involvement and child development, there is a paucity of literature on research specific to fatherhood programming and its impact on the health status of Native men (39–41). Native culture has always known that community health is the sum of its parts, which includes healthy fathers and strong societies (20). Furthermore, it is critical to acknowledge how settler colonization of the Americas have impacted Indigenous masculinity and how this has led to toxic and unhealthy coping mechanisms. This study is an opportunity to engage Native fathers in leveraging their strengths to improve their parenting, economic stability, relationships and, by extension, their overall health status and that of their children. Ultimately this intervention is designed to support Native fathers in the restoration and strengthening of Native societies and the sacred bond between them and their children.

The social, structural, and Indigenous determinants of health affecting Native men and fatherhood serve as a conceptual framework for this study, which aims to: address the aforementioned gap in research by developing and implementing a culturally grounded health intervention to increase economic stability, promote positive parenting, and build healthy relationships among Native fathers. The program is called Azhe'é Bidziil (“Strong Fathers”) and this study protocol illustrates a community-engaged approach to developing and implementing a fatherhood intervention with fathers in two Diné tribal communities. This study is also a direct result of a 30-year community-academic partnership between JHU and the Navajo Nation. Azhe'é Bidziil capitalizes on lessons learned from our family and child interventions, as well as best practices cited in the literature and a community-engaged approach to curriculum development (18, 39).

Study aims are to: 1) to assess the acceptability, feasibility, and satisfaction of Azhe'é Bidziil program among participating fathers; and 2) assess through a pre- post-study design the preliminary impact of the Azhe'é Bidziil program on father involvement, quality of (co-)parenting communication, healthy relationships, fathers' engagement and communication with their children, protective factors (i.e., cultural connectedness and educational/career aspirations), and economic empowerment and stability. The long-term goal of this study is to decrease the number of rural Native families living in poverty, increase the economic stability of Native fathers and their families, reduce violence in rural Native communities, promote positive parenting practices, and increase healthy relationships in Native communities. This study will be conducted with adult (18 years of age and older) Native fathers' and/or father figures (grandfathers, uncles, brothers, etc.) who have at least one child under 24 years of age and reside in or near the Navajo Nation.

Primary research questions are: Is the Azhe'é Bidziil intervention acceptable to Native fathers? Is it feasible to implement? and Are participants satisfied with intervention content and participation? Secondary research questions are: Does the Azhe'é Bidziil intervention have short-term impacts on father-child relationship, self-efficacy, self-esteem, cultural connectedness, psychosocial functioning, economic empowerment, co-parenting, coping skills, healthy relationships and communication, parenting practices, substance use behaviors, and program knowledge?

## Methods and Analysis

### Overview and Hypotheses

We will conduct a pre- post-study to assess preliminary impacts of the Azhe'é Bidziil intervention on risk and protective factors associated with responsible parenting and, secondarily, the feasibility, acceptability, and satisfaction with the intervention. Azhe'é Bidziil consists of twelve sessions which aim to promote fatherhood involvement, responsible parenting, healthy relationships, and economic stability. Study aims and hypotheses are based on the extant literature presented and summarized in the Introduction. Study aim 1 is to assess the acceptability, feasibility, and satisfaction of Azhe'é Bidziil program among participating fathers. The study team hypothesizes that fathers will report high rates of satisfaction with intervention content and their participation and that the intervention and facilitators are culturally and contextually acceptable. We also hypothesize that the intervention is feasible to implement. Study aim 2 is to assess the preliminary impact of the Azhe'é Bidziil program on father involvement, quality of (co-)parenting communication, healthy relationships, fathers' engagement and communication with their children, protective factors (i.e., cultural connectedness and educational/career aspirations), and economic empowerment and stability. The study team hypothesizes that there will be an increase in fathers' positive parenting practices, engagement and communication with their children, cultural connectedness, parenting self-efficacy, and economic empowerment. The study will be conducted in two Diné communities in partnership with JHU. The study was approved by the Navajo Nation Health Research Review Board, local tribal governments, as well as the JHU Institutional Review Board.

### Intervention Adaptation and Development

**Figure 1:**
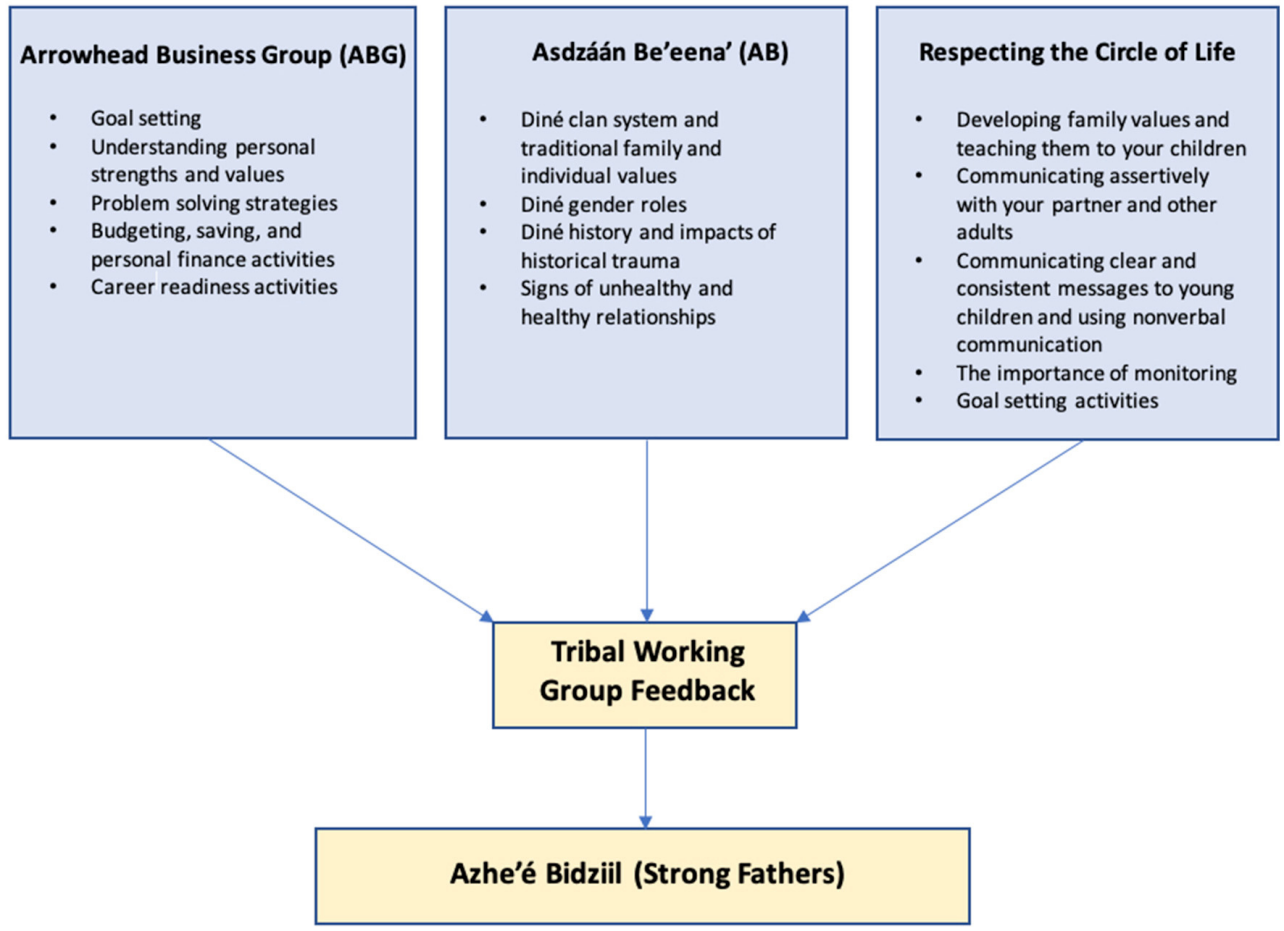
Curricula adaptation process.

Azhe'é Bidziil was adapted from three evidence-based interventions (EBI) developed and proven by the Center (Figure 1). These include: Respecting the Circle of Life (RCL), Arrowhead Business Group (ABG), and Asdzáán Be'eena' (AB). These interventions were selected because they are evidence-based, responsive to locally identified needs, exemplary of our tribal-academic partnership, and were developed by the Center in partnership with tribal communities to be culturally and contextually congruent. In addition, the three interventions have shown positive change in parents and fathers, and cumulatively, constitute the Azhe'é Bidziil curriculum focusing on responsible parenting, promoting and sustaining healthy relationships and marriage, and economic stability.

RCL is a sexual/reproductive health promotion program adapted for Native communities designed to promote general life skills such as goal setting and problem solving while teaching core concepts related to sexual and reproductive health including healthy relationships (42, 43). The program was developed for and evaluated with youth ages 11–19 and a trusted adult who is ≥18 years of age. The first 8 lessons are taught to Native youth in a peer-group camp-based format, and 1 follow-up lesson is taught one on one with youth and their trusted adult at home (42, 43). In a recent randomized controlled trial (RCT) of RCL conducted with *N* = 1,064 Native youth and trusted adults, 12 months after program completion, fathers randomized to the RCL group reported significantly more parent-child engagement (*p* = 0.0163) compared to fathers in the control group (43). Additionally, males receiving RCL reported significantly better partner communication/negotiation skills (*p* = 0.032), connection with their family (*p* = 0.057) and talking with their parent (*p* < 0.001) than those in the control (43). Further, over 85% of participants stated RCL “helps them make better decisions” and 70% reported it “made it easier to communicate with others” (43).

The ABG curriculum consists of 16 lessons and is designed for implementation in high school classrooms with youth ages 13–16. The ABG curriculum focuses on developing personal skills and providing opportunities for Native youth to achieve economic stability with a primary focus on entrepreneurship education and economic security (44–46). ABG teaches essential soft skills necessary for participants to be successful in their relationships (e.g., problem solving, communication, decision making, emotion regulation); provides job skills (e.g., resume building, public speaking, interviewing), training, and goal setting to prepare individuals for future employment; and teaches personal finance and entrepreneurship education to foster a sense of self and upward economic mobility (44). Previous trials of the ABG program indicate it reduces substance use and improves economic stability (45, 46). Specifically, ABG was evaluated through an RCT with N = 393 Native youth and young adults (45). Two years after program completion, ABG participants had significantly higher entrepreneurship knowledge and reported significantly more economic abilities (*p* ≤ 0.0001), economic agency and participation (*p* ≤ 0.0001) and economic confidence and security (*p* = 0.013) compared to controls. They also reported higher levels of future planning and aspiration (*p* = 0.001) as well as intentions to preserve health (*p* = 0.006) (45).

AB is a culturally-grounded program delivered to Native girls and their female caregivers. The overarching goal of the program is to reduce substance use and teen pregnancy by improving caregiver-youth relationships, promoting pride in girls' identity as Native women, improving support systems, and overall improving the psychosocial health of the girl and their caregiver (47). AB consists of 11 lessons, of which 6 are individual lessons taught to the female caregiver and youth, and 5 lessons are group-based lessons taught to all female caregivers and female youth enrolled (47). In a previous pre- post-trial of the AB program at 3 months post-program completion girls and mothers reported improved parent-child relationships compared to baseline (*p* = 0.03 and *p* = 0.004). Girls also had reduced internalizing and externalizing behaviors (*p* ≤ 0.001 and *p* = 0.05), increased self-esteem (*p* = 0.02) and improved social support (*p* = 0.04) (47).

Several components of the RCL, ABG and AB programs were incorporated into Azhe'é Bidziil, but first adapted to fit the needs of Native fathers (Table 1). In addition to incorporating previous EBI content, we adapted the curricula according to best practices for teaching to an older, male-only population. Evidence suggests that adults learn best in more collaborative environments in which they have greater control over the content of the lessons (48). When it comes to learning about gender equity issues specifically, qualitative data from several studies have shown that adult males learn best when they are taught by facilitators who encourage questions and discussion about sensitive topics without judgement or fear of being ridiculed (49). In addition, adult learners show a higher retention rate when they are actively engaged in lessons and they have both extrinsic and intrinsic motivations for learning (48). Engagement in lessons can be achieved by limiting lessons to 2.5 h or less (49). Reflecting on this evidence, we ensured the following: (1) the tone of the curricula presents a conversational, discussion-driven style; (2) lessons were drafted that intentionally engage participants in discussions around sensitive topics like toxic masculinity, domestic violence, and child abuse; (3) to promote honest and direct dialogue, males from the community who have shared life experiences with the participants were recruited and trained to deliver the program; (4) to promote engagement, we limited dependence on technology and classroom-style instruction so the curricula can be facilitated in a variety of community settings (i.e., gymnasiums, outdoor recreational facilities); (5) the time allotted for each lesson was flexible to promote discussion while aiming to keep each lesson under 2 h; (6) finally, we designed lessons to be delivered to groups of men who would stay together for all lessons through a cohort to foster collaboration and peer-to-peer bonding.

**Table 1:** Integration of content from previous EBI.

**Azhe'é Bidziil workshop**	**Curricula used in adaptation**	**Parts of curricula adapted**
1: Striving for a positive future	ABG	• Identifying personal strengths activity • Differentiating values and goals activity • Goal setting activity
	AB	• Diné clan characteristics • Identifying Diné clans activity • Values of the Diné Individual handout
2: Honoring our role as fathers	AB	• Milestones in a man's life in Diné culture • Important roles of men in Diné culture
	RCL	• How to develop family values and teaching them to your children
3: Effective communication and problem solving using the “SPIRIT” approach	ABG	• SPIRIT: A Problem Solving Technique activity
	RCL	• Being Assertive activity • How to communicate clear and consistent messages to young children • The importance of nonverbal communication when communicating with children
4: Boys to men	None	N/A
5: Love does not hurt	AB	• Incorporating the idea of Hózhó and domestic violence. Hózhó means living in balance (health, harmony and beauty). Violence can disrupt Hózhó, and we must take action to regain balance and harmony in our lives. • Traditional roles of women in Diné culture and content related to how Diné people have always held women as sacred.
6: Building healthy relationships	AB	• Signs of healthy and unhealthy relationships • Content on K'é, the Diné kinship system that also includes the idea that in being related to each other, we are responsible to each other, that it is a call to duty for us to respect one another in a loving way.
7: Our history, our future	AB	• Content on Diné history, specifically the Long Walk and boarding schools • Content on historical trauma
8: Monitoring and spending time with your children	RCL	• Content from the parent lesson about the importance of monitoring your children and strategies for communicating with children
9: Basics of budgeting	ABG	• Creating a monthly budget activity • Content about savings and debt, including how to set savings goals and tackle debt
10: Being me and establishing my career	ABG	• Content on the importance of education attainment and salary • Resume building activities and content
11: Career resources	ABG	• Editing a resume activity • Content on strategies for searching and applying for jobs
12: Bringing it all together	RCL	• Making Your Dream Come True goal setting activity • Content on overcoming obstacles on the way to our goals • Compliments and positive messages activity

*Previous EBI: AB, Asdzáán Be'eena'; ABG, Arrowhead Business Group; RCL, Respecting the Circle of Life*.

To assist with adapting these curricula and to ensure relevance and engagement for fathers on the Navajo Nation, we formed a tribal working group consisting of 20 community leaders, local fathers, and researchers with experience working with Native adult males. This working group met 10 times from December 2020 to April 2021. The working group reviewed each of the 12 draft workshops in detail to ensure that the content is culturally appropriate, relevant, and that participants would engage with and understand the information (Figure 1). The members of the working group also proposed additional tailoring, such as providing examples of budgeting for different types of employment such as seasonal work, to strengthen the curriculum, as well as providing ideas to maximize engagement and incentives for participants. The working group also made a critical cultural adaptation by broadening the eligibility criteria to include grandfathers, uncles, stepfathers, and other father figures. This adaptation acknowledges that Native parenting expands beyond the biological parents to include other family caregivers and role models.

The working group was fundamental in helping the team incorporate cultural teachings into the curriculum. Working group members with deep cultural knowledge proposed integrating aspects of Diné culture such as the clan system and the impact of historical trauma to enhance engagement with the curriculum for participants (Table 2). During one working group meeting, one member remarked, “The way I look at it, if you want to have a watered-down curriculum you can focus on modern fatherhood. But I think if you focus on traditional values as the core base of your curriculum it can be an interesting intervention program.” The feedback from the working group was incorporated by two curriculum writers into a comprehensive curriculum that is grounded in Diné values such as respect, reciprocity, discipline, and relationship (50).

**Table 2:** Intervention lesson topics.

**Lesson number**	**Delivery timepoint**	**Cumulative hours**	**Topic**
1	Within 2 weeks of enrollment (week 1)	2	Striving for a positive future: Program introduction, K'é (discuss the meaning of the clan system and stablishing kinship/identity), defining values and goals.
2	Week 2	4	Honoring our role as fathers: Discussing various parenting styles and positive parenting practices, reflecting on cultural role as fathers, reflecting on how own childhood influences parenting style. Developing parenting values.
3	Week 3	6	Effective communication and problem solving: Learn and practice applying the SPIRIT model of problem solving
4	Week 4	8	Boys to men: Understanding masculinity and learning to take care of yourself so you can take care of others.
5	Week 5	10	Building healthy relationships: Foundations of a healthy relationship, enhancing family relationships, skills to strengthen and manage relationships with family members, the benefits of two-parent households (within Native context) and co-parenting strategies and skills.
6	Week 6	12	Our history, our future: Understanding the role of historical trauma, controlling aggressive behaviors and conflict resolution, causes of and addressing child abuse, and nurturing fatherhood practices
7	Week 7	14	Love Doesn't Hurt: Causes of domestic violence/intimate partner violence, understanding the cycle of abuse and seeking help
8	Week 8	16	Monitoring and spending time with your children: Understanding child development and behaviors, strategies to communicate with child throughout development phases, setting limits, and being an involved parent
9	Week 9	18	Basics of budgeting: Difference between wants and needs, building skills and knowledge to improve economic stability, finance management
10	Week 10	20	Being me and establishing my career: Career counseling/development, importance of education and an introduction/creating a resume
11	Week 11	22	Career resources: Resume workshop (continued), industries and opportunities for jobs in the local community, learning to search for and apply for jobs, guest speaker from local employment agency
12	Week 12	24	Bringing it all together: Staying on track and free from substances, how to readjust goals, making a difference in your community, group members give positive feedback to each other.

### Azhe'é Bidziil Intervention

The Azhe'é Bidziil intervention is composed of 12 weekly group sessions (Table 2) conducted with fathers (or father figures such as grandfathers, uncles, stepfathers, and adult brothers) that focus on developing knowledge and skills for promoting positive father involvement, economic stability, and healthy relationships. Each of the 12 group lessons, consisting of 8–12 participants per group, lasts approximately 2 hours and is delivered by two trained Diné male facilitators. Facilitators use a curriculum manual to administer the program and participants receive workbooks. Sessions are taught using didactic techniques that includes lectures, games, role-playing, group bonding activities with corresponding materials, posters, videos and handouts. Group sessions take place either at the local JHU study office or in a communal location, such as outdoor fields, school classrooms, hiking trails, gyms, community centers and/or health facilities. Transportation to group sessions and child-care are provided upon request. Fathers enrolled in the program complete the 12, 2-h workshops over the course of 12 weeks for a total of 24 hours of workshop programming (Table 2). Additionally, social support will be provided to each participant while they are enrolled in the Azhe'é Bidziil program. Social support consists of providing referrals to necessary support services in the community based on identified needs and are conducted either by phone, Zoom, or in-person.

All research staff are Diné and employed/trained by the Center. Research staff complete a background check and are extensively trained in human subject's research and the study protocol, policies and procedures prior to any interaction with participants or study data. To ensure the Azhe'é Bidziil program is implemented with high quality and fidelity, all facilitators receive extensive training in curriculum and must pass a proficiency examination with a score ≥85% prior to delivery. In addition, fidelity monitoring of program implementation will be performed on ≥10% of all sessions conducted. Sessions will be randomly selected and observed in person, audio or video recorded. Staff performing fidelity monitoring will complete a feedback form and review it with the facilitator. Additional training will be conducted as necessary.

### Study Site and Participants

Eligible fathers or father figures must be age 18 years or older and have a primary residence in or within approximately 50 miles of the participating Diné communities. Additionally, fathers must be caregivers of at least one child under the age of 24 years. Eligibility criteria was determined in collaboration with the working group to include all cultural definitions of fatherhood (e.g., uncles, grandfathers, stepfathers, etc.). Based on our eligibility criteria, an 18-year-old father of a newborn and an elderly grandfather who is helping to raise his grandchildren (<24 years old) would both be eligible for the intervention. All eligible participants are required to complete informed consent to participate. Participants who are unable to fully commit to the program (i.e., they are unable to complete all sessions and/or assessments or plan to move during the intervention period), unable to review and sign informed consent, or do not meet the criteria are deemed ineligible for participation.

### Recruitment and Consent

Participants are recruited through a number of outlets using a combination of non-probability and snowball sampling. Participants are recruited by social media, word of mouth, community events, local community partners (e.g., family-based programs and services, schools, housing, and tribal college administration), the local tribal radio station and local newspapers. When a potential participant is identified, a study team member will set up an appointment to discuss the program using an approved recruitment script. Following the recruitment script, the study team member completes an eligibility form to determine if the individual meets the inclusion criteria. If the individual is deemed eligible and interested, a study team member will initiate the informed consent process. Study team members are trained to review the consent form section-by-section with the participant to ensure comprehension and to stop intermittently to answer any questions. Study team members also ask the participant to explain the study in their own words to ensure that they understand the purpose of the study, their participation, and the risks and benefits associated with participation. Oral translation into the Diné language is available upon request.

### Sample Size

Our primary outcome for sample size determination is the change in mean score between baseline and post-intervention in the global father responsibility and involvement scale. We will measure this using the Inventory of Father Involvement (IFI) which consists of 26 items and has been utilized in other studies with low-income, minority fathers. Responses are scored on a Likert scale from 0 to 6. The IFI will be collected at baseline and immediate post-assessment. We anticipate that an increase of 0.24 from baseline to the immediate follow-up time point is reasonable. We assume a baseline mean (SD) of 3.19 (1.25) based on findings reported by low-income fathers in a previous study (51). In order to detect a change in 0.24 between baseline and immediate follow-up timepoint, assuming alpha = 0.05, 90% power, we will need a final sample of 440 participants. Based on previous programs with Native adults that our team has conducted in the participating communities and other programs implemented with fathers (39, 43), we anticipate attrition rates of ~40–45%. We will therefore recruit a total *n* = 750 Fathers.

Fatherhood education programs have high attrition rates, cited in the literature as ranging between one-third and one-half (or higher) of all participants (39–41). The study team has reviewed best practices in fatherhood program retention and are implementing community- and culturally-specific methods for addressing these barriers. Based on our experience with community-engaged research in Native communities, we aim to reduce the attrition rate by hiring and training male facilitators, building peer support, providing child-care during workshops as necessary and transportation to and from workshops (as needed), providing social support, providing gift card incentives for completion of assessments, and by offering flexible workshop schedules. We also plan to implement optional outdoor-based activities (including cultural activities) such as hiking, fishing, basic vehicle mechanics, and a men's sweat lodge to further promote participant retention.

### Outcomes

The outcomes for this pre- post-study are acceptability, feasibility, and satisfaction with the intervention as well as father involvement, quality of (co-)parenting communication, healthy relationships, fathers' engagement and communication with their children, protective factors (i.e., cultural connectedness and educational/career aspirations), and economic empowerment and stability. Please (see Table 3) for a description of measures and key constructs to be utilized.

**Table 3:** Azhe'é Bidziil evaluation measures.

**Measures**	**Key Constructs**	**Description of measure**	**Timepoint**				
			**Pre**	**First**	**Every**	**Last**	**Post**
**Outcome assessment**							
Demographics	NA	Questionnaire to assess age, gender, socioeconomic status and living situation	X				
Program Knowledge	RP, FI, HR, ES	24 questions to assess knowledge and comprehension of key curriculum topics. Developed by study team.	X				X
Healthy relationships and help-seeking	HR	4 questions to assess help-seeking, relationships, skills communication and confidence. Adapted from the Good Road of Life Training for Native Men (52).	X				X
Father involvement	FI	26-item scale to assess fatherhood involvement in the areas of discipline and teaching responsibility, school encouragement, maternal support, providing, time and talking together, praise and affection, academic support, and attentiveness. Adapted from the Inventory of Father Involvement (53).	X				X
Parenting and Co-Parenting	FI, HR, RP	20-item scale to assess the quality of co-parenting communication. Adapted from Coparenting Subscales: Parental Component and Quality of Coparental Communication (54).	X				X
Coping and Conflict Resolution	HR	4-item scale to assess brief resilient coping. Adapted from the Brief Resilience Coping Scale (55).	X				X
Skills, Communication, and Confidence	HR, RP	10 questions to assess perceived skills, communication, and confidence around fatherhood and parenting. Adapted from the Good Road of Life Training for Native Men (52).	X				X
Economic Empowerment and Stability	ES	26-item scale to assess economic empowerment in the areas of expansion of current economic abilities, economic agency and participation, and economic confidence and security. Adapted from a trial conducted with Apache youth (46).	X				X
Cultural Connectedness	NA	11 questions to assess degree of cultural connectedness. Adapted from the Culture is Prevention project (56).	X				X
Substance use	NA	8-item questionnaire used to screen for substance use (57).	X				X
**Feasibility, acceptability, and satisfaction**							
Workshop Feedback Form		Each form is 5 questions to assess the acceptability, feasibility, and satisfaction of individual workshops. Developed by study team.			X		
Program Feedback Form		12 questions to assess the acceptability, feasibility, and satisfaction of the overall program. Developed by study team.				X	
Workshop Session Summary and Attendance Forms		Each facilitator completes a Session Summary Form after each workshop. Forms ask about length, location, attendance and content covered for each workshop.			X		

*Key Constructs: RP, Responsible Parenting; FI, Fatherhood Involvement; HR, Healthy Relationships; ES, Economic Stability; NA, Not applicable*.

### Data Collection

To assess the primary research question, participants will complete short surveys that ask about program acceptability, feasibility and satisfaction at the conclusion of each workshop session and again upon completion of the entire program. Surveys completed at the conclusion of each workshop session are completed via paper and entered by study staff team members into Research Electronic Data Capture (REDCap™). The survey completed at the completion of the program will be administered via REDCap™. Facilitators will also collect comprehensive process data including but not limited to workshop attendance and referrals provided.

To assess the second research question, data is collected at baseline and immediately (within one month) post-intervention completion through via an outcome assessment. All outcome assessment data are collected in participants' homes or another private location via a tablet using REDCap™. All measure items were selected based on their cross-cultural validity. When possible, selected measure items were drawn from studies that were implemented in Native communities and/or among Native men (Table 3). Selected measures were piloted with five Native fathers who provided written feedback on each question. Edits were made accordingly and outcome assessments finalized. See Table 3 for a summary of measures.

### Analysis

For the outcome evaluation data, we will conduct analyses using Stata, version 17 (58). Bivariate regression analyses using mixed effects models will be used to examine change from baseline to post-intervention in outcome measures. Mixed effects multivariate regression models adjusted for repeated measures with a random effect at the individual and group level will be used to examine significance between time points. We will examine whether the mixed effects multiple regression models also need to be adjusted for group levels.

We will review the session implementation assessments (i.e., Workshop Feedback Forms) for acceptability of each individual session, as well as examining the average scores on each item and outcome domain to determine which items and domains were particularly problematic. We will review overall program implementation assessments (i.e., Program Feedback Form) for acceptability of the entire program. Items or domains with average lower than the midpoint scores will be considered barriers to implementation and the focus of program improvement prior to a fully powered effectiveness trial. Finally, we will review all attendance and workshop session data reported by facilitators to understand average dosage and length of sessions.

## Discussion

This study protocol presents one of the few evaluations of a fatherhood intervention to increase economic stability, promote positive parenting, and build healthy relationships among fathers in rural Native communities. Such a study is sorely needed to address the disparate health disparities perpetuated by structural, social and Indigenous determinants of health that Native men experience today. In designing the Azhe'é Bidziil study, several strengths emerged early in the process. The Azhe'é Bidziil study innovatively drew upon evidence-based curricula, fatherhood program best practices, and cultural perspectives in developing an intervention that is tailored for Native men and their unique lived experiences. This would not have been possible without the commitment and knowledge exchange from our tribal working group composed of community leaders, teachers, mental health providers, peacemaking court advocates, coaches, parents, grandparents, and a traditional healer. Each of these partners were dedicated to ensuring that the resulting intervention simultaneously addressed core lessons around economic empowerment, healthy relationships, and father involvement, while also recognizing the critical importance of anchoring the intervention in Native fathers' lived experiences.

Another strength is that the study incorporates a trauma-informed approach in addressing the determinants of Native men's health, such as helping facilitators to reflect on their own understanding of trauma, while also providing tools that aim to advance fathers' social and economic opportunities such as budgeting, career planning, and goal-setting. This approach allows flexibility in gauging and responding to sensitive discussions. For example, in discussing trauma with participants, the facilitators often shift to land-based learning, which acknowledges that land plays “an integral role in Indigenous education” (59). Land-based learning reconnects participants to the land, promotes holistic perspectives, and encourages self-reflection, all of which can be transformative and healing processes (59). Other cultural elements such as Native parenting principles, revitalizing the kinship social support system, and Native gender roles are also unique cornerstones of this study that aim to reinvigorate Native fatherhood practices. The intended benefits of such efforts are 3-fold: cultural connectedness is a known buffer against high-risk behaviors, such as substance use and risky sexual behaviors (60, 61); cultural revitalization is advancement of Indigenous determinants of health which, in turn, promote holistic wellness (15) and cultural elements may promote program retention and enhance social support within groups. Perhaps most importantly, our study's trauma-informed approach to study design included the fundamental community need to have a safe space for Native men to learn, heal, and support one another.

A major limitation of the study is that this is a pre- post-study design and, without a control arm, it is not possible to detect causal relationships between the intervention and changes in key measures. However, it is common practice to have a pre- post-pilot study precede a larger RCT. This practice has its own strengths by allowing time to test curriculum content and to make adjustments prior to a larger scale study. Another limitation of the study is that all measures are self-report, including father involvement, quality of co-parenting relationship, and economic stability measures. Although self-report is commonly used with such study designs and in this specific population, biases are possible. The study employs the REDCap™ virtual data collection and management platform since a similar platform was shown to mitigate bias in another study conducted by JHU with a similar population (62). Another limitation is the piloting of the program in a single region, which means that results are specific to the Diné and nearby tribes and may not apply to other tribal nations. However, we are conscious of the widespread need for an intervention tailored for Native fathers. As such, our curriculum team has made efforts to embed cultural elements that are applicable to most Native nations, such as historical trauma, settler colonialism and masculinity, and extended kinship networks. This includes actively working to bring in Indigenous knowledge systems that focus on gender equality, gender socialization, and traditional gender roles.

If positive results are demonstrated, results from this pre- post-study will inform a large scale RCT of the intervention. Current study findings may also deepen our understanding of peer mentoring, Native men's health status, involvement with their children (especially if they are non-residential), co-parenting relationships, family relationships, cultural connectedness, and economic status. The data collected throughout study implementation may also inform strategies to ensure acceptability, feasibility, and satisfaction of a program designed specifically for Native fathers. Many fatherhood programs experience high attrition and dropout rate, thus if we experience high retention utilizing the aforementioned strategies, our study design could be used to inform other fatherhood program evaluations (39). This study may also provide additional insight into facilitators' proficiencies and trainings needed for program acceptability, feasibility, satisfaction and impact. Our team is consistently working to identify and address training needs in order for male facilitators to teach the curriculum and lead challenging conversations with study participants. Thus, far, this has included providing additional training on toxic masculinity, intersectionality, sexuality, cultural gender roles and responsibilities, and land-based learning strategies. This component has been found to be essential in helping male participants to understand how critical they are to addressing health inequity and restoring cultural practices.

Overall, results from this study may be used to inform future Native fatherhood and Native men's health initiatives and may have far reaching practice, research, and policy implications. It is our hope that this study is a catalyst for future community-academic partnerships that support Native fathers in the strengthening of Native societies and incorporating cultural knowledge to restore the sacred bond between Native fathers and their children. Balance across genders in Native communities is essential for harmony and community mastery. This study presents a unique public health approach that directly addresses the harmful effects of settler colonialism and toxic masculinity amongst Native men while also contributing to the cultural resurgence of Native gender systems.

## Data Availability Statement

The original contributions presented in the study are included in the article/Supplementary Material, further inquiries can be directed to the corresponding author/s.

## Ethics Statement

The studies involving human participants were reviewed and approved by Johns Hopkins University IRB, Navajo Nation Health Research and Review Board. The patients/participants provided their written informed consent to participate in this study.

## Author Contributions

JR, LT, RC, HP, and JM contributed to the conception and design of the study. LG, NT, JB, LS, and BH implemented the study. JR wrote the first draft of the manuscript. LT, TB, HP, and JM wrote sections of the manuscript. All authors contributed to manuscript revision, read, and approved the submitted version.

## Conflict of Interest

The authors declare that the research was conducted in the absence of any commercial or financial relationships that could be construed as a potential conflict of interest.

## Publisher's Note

All claims expressed in this article are solely those of the authors and do not necessarily represent those of their affiliated organizations, or those of the publisher, the editors and the reviewers. Any product that may be evaluated in this article, or claim that may be made by its manufacturer, is not guaranteed or endorsed by the publisher.
