# Supplementary material for: Azhe'é Bidziil (Strong Fathers): Study Protocol for the Pilot Evaluation of an American Indian Fatherhood Program to Improve the Health and Wellbeing of Diné (Navajo) Fathers
Source: Front Public Health. 2022 Feb 10;9:790024. doi: 10.3389/fpubh.2021.790024 (PMC8867173; doi:10.3389/fpubh.2021.790024)
Supplement: Supplementary file 1 [file Table_1.DOCX]

**Table 1. Inclusion and Exclusion Criteria**

| **Inclusion criteria** |  |
| --- | --- |
|  | Adult male father or father figure > 18 years of age |
|  | Caregiving for a child <24 years of age |
|  | Residence on or near the reservation community |
|  | Complete all assessments and attend 12 weekly group sessions |
|  | Review and sign an informed consent |
| **Exclusion criteria** |  |
|  | Unwilling to participate in the full program |
|  | Unwilling to complete assessments and attend group sessions |
